# An Innovative Approach to Control Steel Reinforcement Corrosion by Self-Healing

**DOI:** 10.3390/ma11020309

**Published:** 2018-02-20

**Authors:** Dessi A. Koleva

**Affiliations:** Faculty of Civil Engineering and Geosciences, Department 3MD, Materials and Environment, Delft University of Technology, Stevinweg 1, 2628 CN, Delft, The Netherlands; d.a.koleva@tudelft.nl; Tel.: +31-1527-87451

**Keywords:** corrosion, reinforced concrete, polymeric nanomaterials, self-healing

## Abstract

The corrosion of reinforced steel, and subsequent reinforced concrete degradation, is a major concern for infrastructure durability. New materials with specific, tailor-made properties or the establishment of optimum construction regimes are among the many approaches to improving civil structure performance. Ideally, novel materials would carry self-repairing or self-healing capacities, triggered in the event of detrimental influence and/or damage. Controlling or altering a material’s behavior at the nano-level would result in traditional materials with radically enhanced properties. Nevertheless, nanotechnology applications are still rare in construction, and would break new ground in engineering practice. An approach to controlling the corrosion-related degradation of reinforced concrete was designed as a synergetic action of electrochemistry, cement chemistry and nanotechnology. This contribution presents the concept of the approach, namely to simultaneously achieve steel corrosion resistance and improved bulk matrix properties. The technical background and challenges for the application of polymeric nanomaterials in the field are briefly outlined in view of this concept, which has the added value of self-healing. The credibility of the approach is discussed with reference to previously reported outcomes, and is illustrated via the results of the steel electrochemical responses and microscopic evaluations of the discussed materials.

## 1. Introduction

Reinforced concrete is a durable material, capable of withstanding a variety of adverse environmental conditions. It is a highly alkaline composite, where the pH of the pore solution ranges between 12.7 and 13.5. The cementitious matrix in reinforced concrete acts as a physicochemical barrier and assures the passivity of the steel reinforcement, hence it usually provides corrosion protection for the steel surface. However, the open pore structure of the concrete cover (and matrix), allows aggressive substances to penetrate into the bulk material, initiating steel corrosion. The onset of steel corrosion is due to carbonation (a pH drop < 9 in the bulk matrix) or chloride contamination (resulting in a corrosion-related local pH drop < 5 on the steel surface) [1,2,3,4,5,6,7]. Upon steel de-passivation, corrosion initiation and propagation take place, increasing in rate, ultimately causing concrete cracking as a result of rust expansion. Concurrently, the steel cross-section is reduced, increasing the potential risks of a rapid structural failure.

So far, various mitigation and protection techniques have been investigated and applied, e.g., corrosion inhibitors, [8,9,10], protective (including polymer) coatings, surface sealers, etc. [11,12]. Among the electrochemical techniques for corrosion control, impressed current cathodic protection (ICCP) is known to be applied to structures in highly aggressive environments [5]. However, ICCP only targets the steel reinforcement and is well known to result in side effects for the bulk matrix or the steel/cement-paste interface, for instance an alkali aggregate reaction and/or bond-strength degradation [13,14,15,16,17,18]. Together with the risks of hydrogen embrittlement for the protected steel in pre-stressed concrete [14,15,16], ICCP has drawbacks and limitations for certain classes of material. Other electrochemical techniques, e.g., desalination and re-alkalization, conversely only target the cement-based material, restoring the chemical composition of the concrete pore water [16]. The result is a favorable medium for potential steel re-passivation. In other words, the available methods for corrosion control target either the steel alone, or only the concrete material.

Novel solutions for new cement blends, steel grades, novel coatings, self-healing approaches, etc., have been reported to show great potential [19,20,21,22,23,24,25,26,27]. Nevertheless, these methods, although claiming corrosion delay as a final outcome, aim only at the quality of the cement-based material. They do not consider the overall complex electrochemistry that governs the corrosion process itself or the phenomena within corrosion control. For instance, the involvement of nanomaterials (e.g., polymeric nanoparticles, inorganic nanoparticles, etc.) in cement-based systems was reported to result in increased compressive strength, matrix densification, resistance to the penetration of aggressive substances, etc. [28,29,30]. A comprehensive review of nanotechnology in concrete material science [31] gives a detailed overview of the application of nanomaterials for altered properties of cement-based systems. The majority of the literature reports, however, deal separately with either only the cement-based bulk matrix or the steel surface.

In view of the application of polymeric additives, including polymeric nanoparticles, in (reinforced) cement-based materials, admixed polyethylene oxide (PEO)-based polymers were reported to result in the re-distribution and improved dispersion of hydration products, an increase of electrostatic repulsive forces, and improved microstructural properties [32,33]. Here again, the target is only the cement-based material. Additionally, these admixtures are used in comparatively large quantities, e.g., between 3 wt % and 30 wt % per dry cement weight, as opposed to the possibility of employing significantly lower amounts of, for instance, polymeric nanomaterials. The commercially available “XSeed”, a polymer-coated calcium–silicate–hydrate (CSH), is reported to be used in a concentration of 0.3 wt % per cement weight, improving microstructural properties [24]. All the above admixtures are used in significantly higher amounts if compared to the potential of employing tailor-made polymeric nanomaterials, e.g., at a minimum ten times lower concentration, as with the nanoparticles suggested in this work.

Besides the uses mentioned above, the application of polymeric nanoparticles for simultaneously achieved corrosion protection and improved bulk matrix properties in reinforced concrete has, to the author’s best knowledge, not been reported by other authors or research groups. This is where this work aims at raising awareness regarding a feasible approach, which targets the reinforced concrete system as a whole, emphasizing the application of polymeric nanomaterials for proactive corrosion control in civil structures.

A brief background on steel passivity, passivity breakdown and cement-matrix-related degradation mechanisms are outlined first in view of the subject of this work. Next, the detailed concept of the above approach is communicated. The credibility of the approach is discussed with reference to previously reported outcomes. As concept-supporting information, the results for the steel electrochemical response, together with microscopic observations regarding the discussed materials and interfaces, are also presented.

## 2. Steel Passivity, Passivity Breakdown and Matrix Carbonation—Background

### 2.1. Steel Passivity in Reinforced Concrete

Corrosion of the steel reinforcement in reinforced concrete is an electrochemical corrosion process. Although concrete is referred to as a solid material, the pore water in the concrete bulk matrix is an aqueous medium. Hence, the pore water in contact with the steel reinforcement allows for oxidation and reduction reactions on the steel surface to be initiated and to progress over time. In a simplified way, the corrosion of steel in reinforced concrete is governed by two electrochemical reactions, presented by Equations (1) and (2), i.e., oxidation, or the dissolution of iron at anodic sites (Equation (1)) and the reduction of oxygen at local cathodes, consuming electrons generated by the metal dissolution (Equation (2)). The products of these reactions interact and in a final stage transform into a stable protective film on the steel surface. This protective (passive) film remains intact if not disturbed by aggressive substances (e.g., chloride ions) and/or changes in the pH of the concrete pore water (e.g., due to matrix carbonation). In the simplest case, a two layer structure of the passive film can be assumed, where the inner layer of Fe3O4 adheres well to the steel substrate, restricts subsequent film growth and, therefore, limits further oxidation [34].

Passivity is often assigned to the outer, gelatinous layer of hydrous Fe_2_O_3_, however, both oxides constitute the protective ability and properties of the passive film on the steel surface. In other words, the oxidation and reduction processes (Equations (1) and (2)) are followed by film precipitation and development, for instance, the chemical reaction mechanisms as given in Equations (3) and (4), would initially take place. This film is a combination and/or a predominant occurrence of ferrous, Fe^2+^ and/or ferric, Fe^3+^ based compounds.

2Fe → 2Fe^2+^ + 4e^−^ (1)

O_2_ + 2H_2_O + 4e^−^ → 4OH^−^ (2)

Fe^2+^ + 2OH^−^ → Fe(OH)_2_ (3)

Fe(OH)_2_ + ¼ O_2_ → γ-FeOOH + ½H_2_O (4)

In the absence of chlorides and with the sustained pH of the medium, both compounds are chemically stable in the alkaline medium of the concrete matrix. With the increase in the maturity of the system overall, and depending on oxygen availability, the ferrous oxides tend to convert to more stable ferric oxides.

### 2.2. Passivity Breakdown

In conditions when chlorides are present in the pore water, the cement layers adjacent to the steel surface, or in the case of matrix carbonation (CO_2_ penetration, resulting in a pH drop of the pore water), soluble complexes are formed from the originally available ferrous oxides. For example, intermediate compounds such as green rusts of type I and II (Equations (5) and (6)) will be formed. These are not-protective and do not constitute an adherent layer. Additionally, green rusts, as well as Fe(OH)_2_, are known to contain both hexagonal and cubic layers of close-packed oxygen [35]. These compounds exist prior to the formation of the purely cubic close-packed structure of the stable end products, i.e., Fe_3_O_4_ (FeO + Fe_2_O_3_), γ-FeOOH or γ-Fe_2_O_3_.

6Fe(OH)_2_ + CO_3_^2−^ + 2H_2_O → [Fe_4_^2+^ Fe_2_^3+^(OH)_12_][CO_3_·2H_2_O] + 2e^−^ (5)

4Fe(OH)_2_ + Cl^−^ → [Fe_3_^2+^ Fe^3+^(OH)_8_Cl] + e^−^ (6)

In the presence of chloride ions, the stability of a close-packed arrangement is higher [36]. Consequently, a steel surface covered by a ferric-oxide-based film (Fe^3+^-based) will exhibit a higher resistance to (electro) chemical reaction mechanisms involving carbonate or chloride ions, and subsequently, higher resistance to corrosion initiation and propagation [37].

According to existing models for chloride-induced passivity breakdown, an initial adsorption of chloride anions on the oxide (passive) film takes place [38], resulting in its chemical dissolution. This mechanism is very local in character [39] and initiates predominantly on structural defects or inhomogeneities on the steel surface [40]. The adsorption of chloride anions enables new pathways for charge exchange, which allows for steel corrosion initiation and/or propagation on the steel surface [39]. This mechanism of passivity breakdown, and the further increase in corrosion rate due to chloride ions in the medium, is similarly relevant for reinforced concrete. Free chloride ions only, or those present in the pore solution, would initiate or participate in the passivity breakdown and subsequent steel corrosion process. The ability of the concrete matrix to chemically bound chlorides (or the chloride-binding capacity of cementitious materials, and the formation of, e.g., calcium–chloro–aluminates) is, in fact, one of the reasons for the initial “delay” of chloride-induced corrosion in reinforced concrete, even when the penetration of a substantial amount of chloride ions from, e.g., the external environment, does exist. Therefore, the term “chloride threshold” has been accepted in the field, linking the chloride content at the time of corrosion initiation [41,42,43,44,45]. This aspect is not subject to further elaboration in this contribution, but mentioned for the clarity of the discussed mechanisms.

### 2.3. Matrix Carbonation—Steel-Corrosion-Related Aspects

Chemically bound chlorides can participate in the corrosion process if a pH drop in the pore solution occurs, as in the case of carbonation, for example. Carbonation is the process by which atmospheric CO2 slowly propagates in the depth of the bulk concrete. According to the simplified sequence of a carbonation process, CO_2_ penetrates the concrete cover, dissolves in the pore solution and reacts with Ca-bearing phases, e.g., Ca(OH)_2_, silicates and aluminates, which are all constituents of the cementitious bulk matrix. Chemical reactions, as the ones presented by Equations (7)–(10), would take place:

Ca(OH)_2_ + CO_2_ → CaCO_3_ + H_2_O (7)

2SiO_2_·2CaO·2H_2_O + 3CO_2_ → 2SiO_2_ + 3CaCO_3_ + 3H_2_O (8)

4CaO·Al_2_O_3_·13H_2_O + 4CO_2_ → 2Al(OH)_3_ + 4CaCO_3_ + 10H_2_O (9)

CaCO_3_ + H_2_O + 2CO_2_ → Ca(HCO_3_)_2_ (10)

These reactions, (Equations (7)–(10)), lead to a pH drop in the pore solution from the original pH of ≥ 12.7 to pH of ca. 8 [37,46]. Excess CO_2_ in the pore water results in the formation of bicarbonate (Equation (10)), additionally lowering the pH of the pore solution. This leads to the dissolution of Ca-bearing phases (Equations (7)–(9)), and consequently, also to the dissolution of calcium–chloro–aluminate complexes, which otherwise chemically bind chloride ions. Along with local acidification, the result is an increase in the free chloride ion concentration.

Besides resulting in changes of the pH of the pore water, the dissolution of Ca-bearing phases due to carbonation exerts alterations in the original matrix composition. Uniform (general) corrosion on the steel surface will be the result of lowering the pH of the pore water. In cases when both carbonation and chloride ingress are at hand, general corrosion would co-exist with chloride-induced, localized corrosion on the steel surface. Here again, the initially chemically-bound chlorides could be released, increasing the chloride threshold level for corrosion initiation [47,48], and/or contributing to an already ongoing corrosion process.

Carbonation of the matrix would also affect the properties of the overall product layer on the steel surface. In reinforced concrete, the protective ability of the passive film is additionally supported by Ca-rich phases, adhering well to the (steel) substrate or accumulated at the steel/cement matrix interface. Some reports discuss only a limited protection efficiency of the adhered calcium-rich layer [49], while others claim an increased protection ability of the Ca-substituted Fe_3_O_4_ and/or Fe_2_O_3_ [34,50]. This layer is already different from the purely passive film, and is, therefore, to be considered as a product layer on the steel surface. The increased protective ability of a Ca-rich, iron oxide/hydroxide layer on the steel surface is due to the incorporation of Ca(OH)_2_ in the outer atom layers of the inner passive film, a consequence of the adsorption of Ca^2+^ ions in this (inner Fe_3_O_4_) film. Consequently, altered Ca-bearing phases in the system overall, or a reduced amount of Ca-containing compounds in the vicinity of the steel surface, as in conditions of carbonation, will result in reduced protective ability of the Ca-substituted iron oxide/hydroxide product layer.

The above considerations are important in view of the concept discussed in this work and its approach to corrosion control, regarding two aspects. First, appreciation of the composition of the passive (and product) layer on the steel surface, when no passivity breakdown is at hand, was considered a useful reminder in view of the concept of corrosion control discussed in this paper. More importantly, the potential for a sustained or improved protective ability of the passive film, or the overall product layer on the steel surface, by, e.g., maintaining the Ca substitution of iron oxides/hydroxides, is of particular interest. Secondly, by considering the structure, composition and protective abilities of the product layer, together with relevant microstructural alterations in the bulk matrix, one can account for the presence of a (potential) excess of certain substances e.g., Ca-bearing compounds. 

## 3. Experimental Program—Introduction to Sequence and Approach

The approach to corrosion control by utilizing specifically chosen nanomaterials was studied in sequential steps and a series of experiments, as normally employed in corrosion and corrosion control studies for reinforced concrete [51,52,53,54,55,56,57,58]. This is especially relevant to cases, as in this approach, where a modified (concrete) mix design and/or admixtures are to be evaluated for their effect on both the corrosion state of the steel reinforcement and the properties of the cementitious matrix. For instance, prior to tests in a steel-reinforced, cement-based system, corrosion studies in model aqueous medium, resembling the concrete pore water, were performed in parallel to studies of plain (non-reinforced) cement-based specimens. This allowed a preliminary evaluation of the effect of, e.g., the admixed nanoparticles in systems with lower heterogeneity levels. Hence, the electrochemical state of the steel surface could be more precisely evaluated, while the micromechanical, microstructural, etc., properties of the cementitious bulk material could be derived without an additional contribution of interfaces (e.g., steel/cement-paste interface). Positive outcomes would justify the next steps of studying the new additives or mix design in a reinforced mortar or concrete system, already allowing for evaluation of the material properties of both the steel and cementitious matrix together. The above considerations were followed with regard to the hereby discussed approach to corrosion control in reinforced concrete using nanomaterials. Consequently, a large number of experimental materials and methods were involved. 

This paper examines only the concept of the approach to corrosion-induced damage control with the added value of self-healing. The experimental results, therefore, are limited to those supporting the credibility of the approach. Hence, details on the materials and methods are outlined at the end of this contribution as supporting information, narrowed down to the necessary minimum, where reference is also made to the full details of each method or experiment. 

## 4. The Concept of Nanoparticle Application in Reinforced Concrete

### 4.1. The Approach to Corrosion Control via Nanoparticles

The approach to corrosion control in reinforced concrete using nanoparticles simultaneously targets the electrochemical response of the steel reinforcement, the material properties of the bulk cementitious matrix, as well as the properties at the steel/cement-paste interface and cement/aggregate (sand or gravel) interfaces. This synergetic approach (involving the fields of cement chemistry, micromechanics, electrochemistry and nanopolymer interactions) aims to reduce bulk matrix and interface permeability and to reduce pore network connectivity, and consequently to minimize diffusivity in the presence of polymeric nanoparticles. These would in turn result in a decreased penetration of aggressive substances in the bulk (cement-based) material. Improved properties of the passive film on the steel reinforcement were expected to be simultaneously achieved, as a result of the presence of the nanoparticles in the vicinity of the steel surface (barrier effects at the very least were expected). Next, only in the event of external influences (such as CO_2_ penetration, hence carbonation in the matrix, or Cl-penetration, followed by localized corrosion on the steel surface) would the nanoparticles participate in a self-healing mechanism. This would be triggered by release of a chosen chemical substance initially incorporated in their interior (e.g., CaO, Ca(OH)_2_). The result would be at least a partially compensated calcium content in the bulk matrix and an improved (or ideally repaired) product layer on the steel surface. 

The above mechanisms are not inhibitive-action-related, e.g., CaO (or Ca(OH)_2_), and do not have inhibitive properties. The Ca-based “self-healing agent”, trapped in the inner volume of the nanoparticles, was chosen as such due to the natural predominance of Ca in cement-based systems, with the following motivation: (i) the cementitious hydration products are Ca-based and possible reduction of the calcium content (e.g., due to carbonation-related phenomena or leaching-out in the presence of concentration gradients) negatively impacts microstructural and mechanical properties [59,60,61,62]; (ii) Ca-substituted iron oxide/hydroxide layers on the steel surface, as previously discussed, are known to be more corrosion resistant ([63], and references therein).

### 4.2. The Concept with the Added Value of the Self-Healing of Corrosion Damage

**Figure 1:**
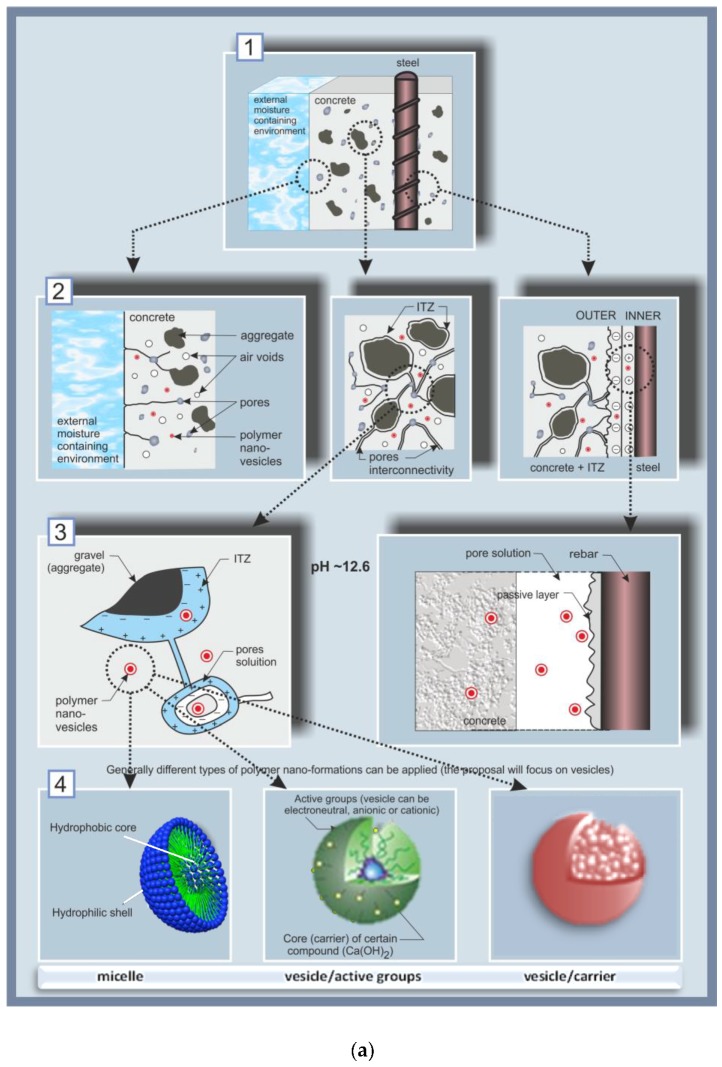

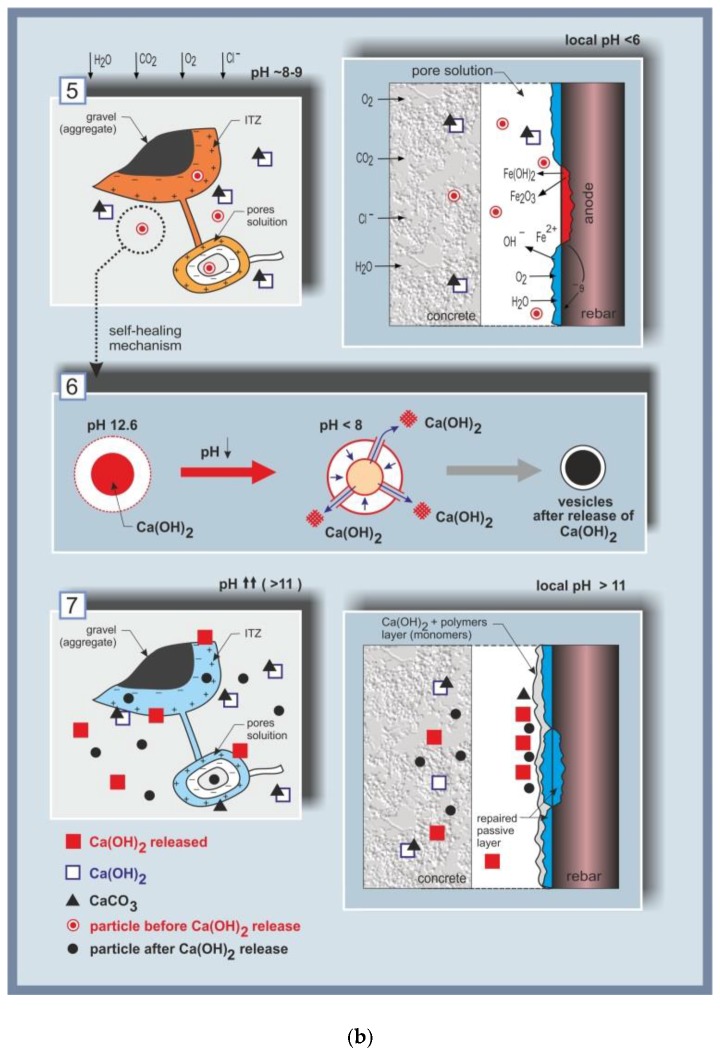
(**a**) The reinforced concrete system, (1), with relevant zones of interest, (2) and (3), i.e., the cementitious bulk matrix, the steel/cement-paste interface, and admixed nanoparticle distribution, (3), together with the relevant types of nanoparticles,(4); *left—*the system prior to changes from external factors, sections (1) to (4); (**b**) The reinforced concrete system after degradation, (5), followed by self-healing, (7), due to the effect of nanoparticles, (6).

The concept of degradation control, with the added value of self-healing in the case of carbonation or chloride-induced corrosion, is schematically presented in Figure 1, from the event of damage initiation to the self-healing mechanism. 

Figure 1a,b include sections (1) to (7), visualizing the main aspects with regard to reinforced concrete as a system, and outlining the approach to the degradation control employing nanoparticles. In Figure 1a, sections (1) to (3) present the reinforced concrete system, zones and interfaces of interest; section (4) includes a schematic presentation of the considered nanoparticles, i.e., polymeric micelles and vesicles. In Figure 1b, sections (5) to (7) schematically present the degradation events, related mechanisms and final outcome. The interfaces of major interest in the system “reinforced concrete” (Figure 1a, section (1)) are depicted in more detail in section (2), Figure 1a, visualizing the interface of the concrete cover/environment (left), the bulk cement-based matrix (middle) and the steel/cement-paste interface (right). The bulk concrete matrix, in the middle of section (2), is presented zoomed-in as section (3) (Figure 1a), presenting a portion of an aggregate particle (e.g., sand) in the cement matrix, the interfacial transition zone (ITZ) of the aggregate/cement paste, including a pore pathway (a connected pore and pore solution), both in the ITZ and the pore.

Figure 1 also schematically presents the expected uniform distribution of nanoparticles in each zone and at relevant interfaces (Figure 1a, sections (2), (3) and (4)). The nanoparticles, section (4), were expected to exert positive effects on both the cement-based material, as well as on the electrochemical state of the steel reinforcement. For instance, in their presence, reductions in porosity and pore size (section (3) (left), Figure 1a) are targeted for the cementitious material and relevant interfaces, on the one hand. On the other hand, the electrical double layer—see Figure 1a, section (3) (right)—and the overall electrochemical state of the steel surface, were expected to contribute to steel corrosion control. These alterations in material properties would be at hand prior to any degradation-related occurrences (e.g., pH reduction of the pore solution due to matrix carbonation or corrosion initiation, following chloride ion penetration). 

Section (4), Figure 1a, schematically presents the particles chosen for this approach, based on polyethylene–oxide–polystyrene co-block polymers i.e., PEOx-b-PSy. Micelles and vesicles were intended for use, both being formations of a hydrophilic outer portion (corona or shell) and a hydrophobic counterpart (the “core” or enclosed volume). The micelles would affect the material properties mainly due to the presence of the polymer, whereas the effect of the vesicles was intended to be both due to the polymer itself and due to release of the loaded compound. In order to distinguish between these two effects, both “empty” vesicles (i.e., just water reservoirs) or “loaded” ones, i.e., with Ca-based compounds entrapped in their interior (Ca(OH)_2_ in this case) were tested later in the experimental program.

**Figure 2:**
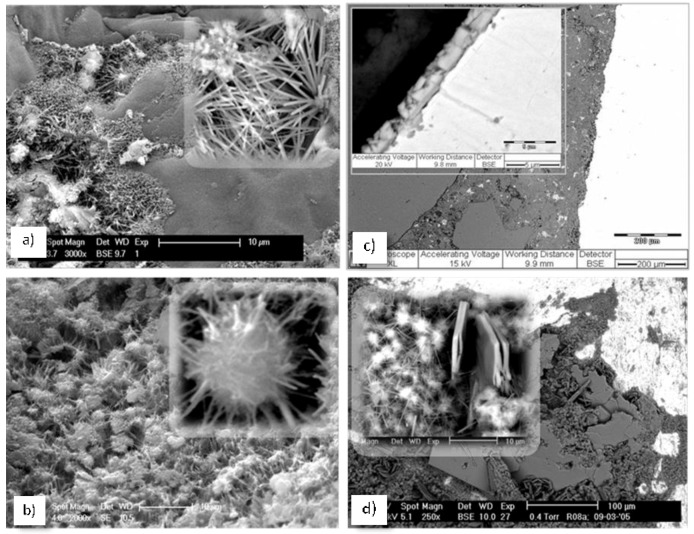
Electron microscopy (ESEM) micrographs, depicting the cement-based bulk matrix (**a**,**b**) and embedded steel (**c**,**d**) as follows: (**a**) a fracture of the interface aggregate (sand particle)/cement matrix, where the inlet depicts a hydration product (ettringite) of needle-shape morphology; (**b**) cement paste bulk matrix only—morphological observations on a fracture surface, depicting calcium–silicate hydrate (CSH) nucleation and growth; (**c**) the steel/cement-paste interface in reinforced mortar (**a** polished cross section), where the following are well visible: the aggregate (sand particles) in the matrix, the cement paste bulk (in the proximity of the steel surface) and a well-adhered product layer (inlet) on the steel surface (the steel reinforcement appears on the right side of the image and in the inlet); (**d**) longitudinal section (top surface) of a steel reinforcement, partially covered with cement relicts and Ca-based compounds of platy morphology (CaO, Ca(OH)_2_), together with CSH particles (inlet).

The interfaces schematically depicted in Figure 1a,b are also shown as experimental results in the Electron Microscopy (ESEM) micrographs in Figure 2 further below. For instance, the interfaces in the cementitious matrix (aggregate/cement paste) as given in Figure 1a, sections (2) and (3) (left and middle) are presented in Figure 2a,b, while the steel/cement-paste interface as sketched in Figure 1a, sections (2) and (3) (right) are depicted in Figure 2c,d, including the product layer on the steel surface (inlet in Figure 2c,d).

When no changes in the environment are relevant, the high alkalinity of the pore solution (pH ~ 12.7–13.5) maintains steel passivity, i.e., the steel surface is covered by a passive layer (Figure 1a, section (3) (right)), including a well-adhering product layer (Figure 2c) and Ca-based surface products—Figure 2d). Together with improved bulk matrix characteristics, the nanoparticles, present in the vicinity of the steel surface, were expected to improve the properties of the passive layer. For instance, enhanced barrier effects would be expected due to physical or chemical interactions of the particles and cement hydration products. Additionally, the nanoparticles would interact with the iron oxide/hydroxide layer on the steel surface, hence also affecting the steel/cement-paste interface (electron microscopy of this interface is illustrated in Figure 2c,d). In other words, the admixed nanoparticles would result in superior material properties when no degradation mechanisms are yet involved.

**Figure 3:**
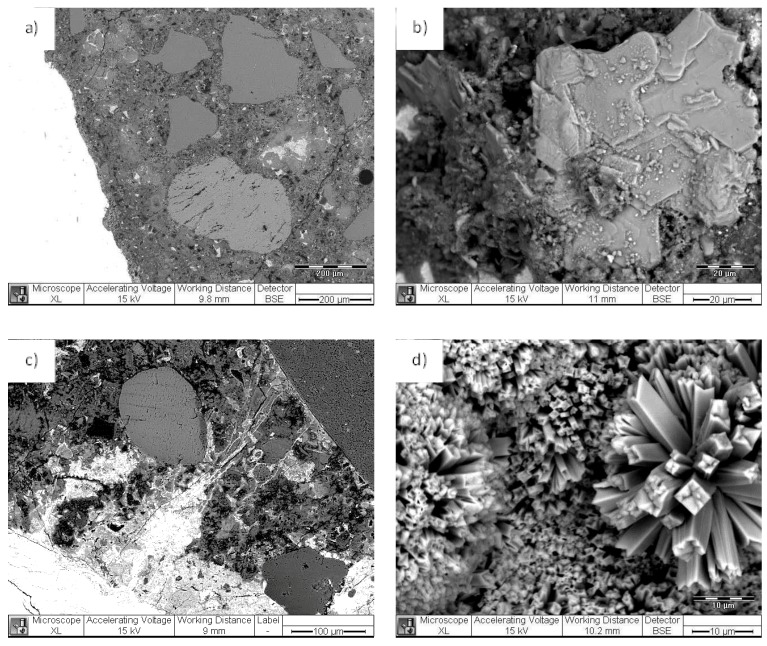
ESEM micrographs: steel/cement-paste interface in reinforced mortar (**a**,**c**) and product layer on the steel surface (**b**,**d**) for a control, non-corroding (**a**,**b**) and a corroding specimen (**c**,**d**).

Section (5) in Figure 1b presents the effect of carbonation (left) or chloride-induced corrosion (right). As previously discussed, carbonation is the process of the reaction of CO_2_ with Ca-bearing compounds in the matrix, i.e., calcium–silica–hydrate (C-S-H) and Ca(OH)_2_, formation of CaCO_3_, which deposits in the bulk matrix, blocking the Ca(OH)_2_ (Figure 1b, section (5) (left)). This leads to a reduction of pH to ca. 8–9 in the pore solution and ultimately to uniform corrosion of the steel reinforcement. On the other hand, chlorides present at the steel/cement-paste interface will induce localized corrosion of the steel reinforcement and break down the passive layer (a local drop of pH to <6 would be relevant on the steel surface and within the localized damage (section (5), Figure 1b, (right), showing the formation of corrosion cells, ionic and electron flow, corrosion product formation, and the re-distribution of anodic and cathodic locations on the steel surface). Typical chloride-induced corrosion damage on reinforcing steel is presented by the ESEM micrographs in Figure 3c,d in comparison to control, non-corroding reinforcement (Figure 3a,b). Both cases in Figure 3 depict the steel/cement-paste interface in reinforced mortar (left) and the product layer on the steel surface (right). These will be discussed further below with respect to the outcomes when employing nanoparticles for corrosion control (Section 5).

Following the concept of the approach as given in Figure 1, the pH alterations in the pore solution, or those at the steel surface, were to trigger re-structuring of the initially admixed nanoparticles, section (6) in Figure 1b. A nanoparticle that releases an entrapped compound using a change in pH as a trigger was the original idea of the approach. However, the employed micelles and vesicles, being Polystyrene (PS)-based, were in fact stable in the event of pH changes. Therefore, the mechanisms upon which the nanoparticles exerted positive effects and improved material properties, finally leading to the self-healing of the previously-induced damage, section (7) in Figure 1b, were mainly mechanisms such as (i) salting-out effects of their PEO portion; (ii) the re-structuring and release of the core-containing compound due to osmotic pressure and micromechanical stress within hydration and corrosion product alterations and/or growth; and (iii) “nucleation site” effects1 prior to degradation phenomena. Additionally the hydrophobic PS-portion of the nanoparticles, which has a high glass transition temperature (Tg), results in the stability of the micelle or vesicle formation in conditions of no internal transformations and, consequently, to a gradual release of any hydrophobic load [64], which would be directed towards a higher release rate in the presence of micromechanical stress. The release of Ca-based compounds from the vesicles, induced by the above triggers, or by de-hydration in the presence of chlorides, will result in partial recovery of the calcium content in the pore solution, while the hydrophobic corona (or vesicle shell) will collapse over the hydrophobic PS counterpart (or the core). The “empty” nanoparticles would still contribute to reducing porosity in the system and bridging gaps or micro-cracks (the concept of which will be illustrated in Section 5), while the released compounds would stabilize the pore solution composition and/or improve the protective ability of the passive and product layers on the steel surface. 

## 5. Credibility of the Concept and Approach

### 5.1. PEO-b-PS Performance in Contact with Cementitious Materials—Preliminary Studies

Prior to the production of PEO-b-PS-based micelles and vesicles for the purposes of the above discussed approach, the performance of this block co-polymer was evaluated when in contact with a cementitious material. Calcium-sulfoaluminate-based expansive additive (CSA) was used for this purpose, where the CSA particles (as dry powder) were “embedded” in a PEO-b-PS film. CSA was chosen for this demonstrative test, since the compound is a highly expansive agent, forming well distinguishable hydration products (ettringite) upon contact with the aqueous medium. This means that upon damage of the PEO-b-PS film, the CSA particles will immediately react to form well-visible hydration products.

The aim of this test was to observe the overall performance of PEO-b-PS in contact with cementitious hydration products. This was important in view of the performance of PEO-b-PS-based micelles or vesicles, when added to a cement-based mixture for the purposes of the approach described above. Next, the aim was to illustrate a designed release of cementitious hydration products when the CSA particles, initially “coated” with PEO-b-PS, would come in contact with alkaline solutions (like the pore water in a cement-based matrix). This process would occur in the event of a mechanical trigger (e.g., a micro-crack in the cementitious matrix) or damage of the PEO-b-PS “coating”. Further, the produced hydration products would fill in gaps or bridge cracks, while the polymer itself would remain within the hydration products. This section only provides a visual illustration of the observed interactions.

**Figure 4:**
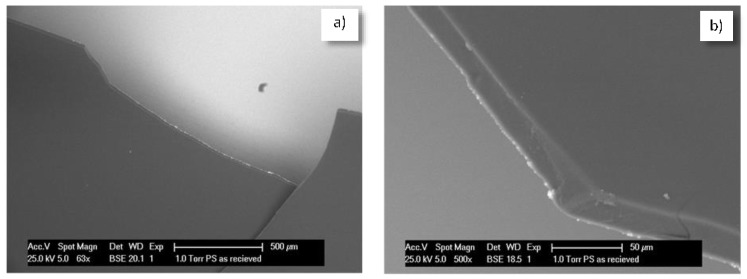
Polyethylene oxide-b-polystyrene coated Calcium sulfoaluminate (PEO-b-PS + CSA) composite—as produced (non-treated) film at magnification 63x (**a**) and 500x (**b**).

A procedure of a “reversed micelle” formation was performed to coat single CSA particles (generally of the size between 10 and 100 micrometers) with a PEO-b-PS film: (i) an organic solvent that would allow the formation of a film but would prevent the initial formation of hydration products was used; (ii) the “coated” CSA was dried-out and produced as a film of individually coated CSA particles. The microscopic investigation in Figure 4 presents the uniform, non-treated film of the CSA+PEO-b-PS composite. The composite was broken into flakes and was water treated for 24 h.

**Figure 5:**
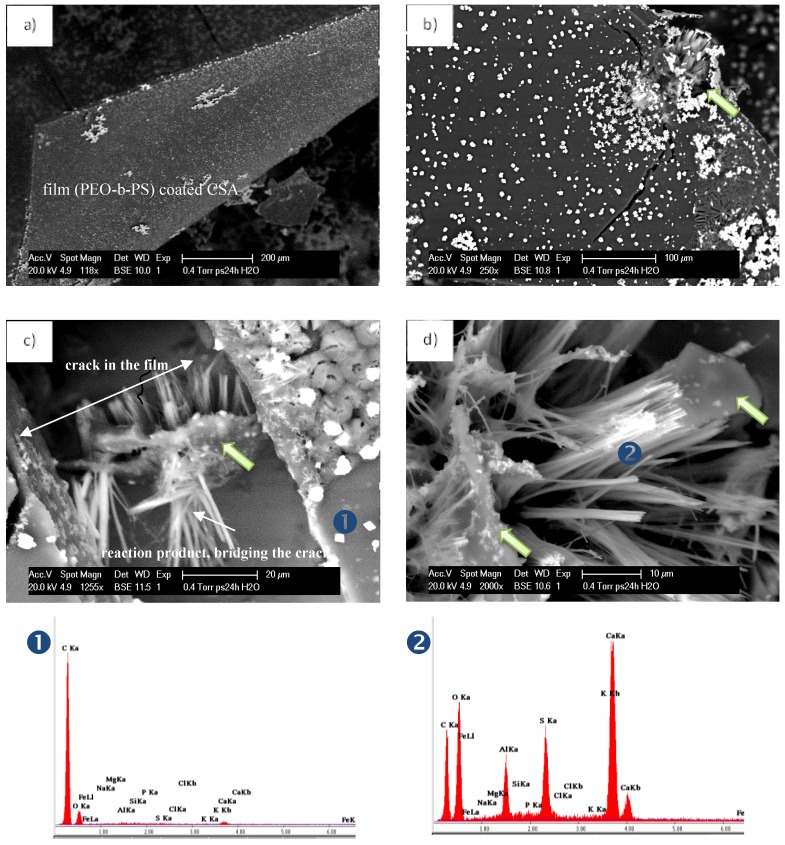
PEO-b-PS + CSA composite after 24 h treatment in aqueous medium (**a**, **b**); higher magnification of the reacted CSA, bridging micro-cracks (**c**) and a remaining PEO-b-PS on the hydration product (**d**). The EDX patterns (**bottom of figure**) present the result from local spot analysis, corresponding to the indicated locations in c) and d).

Figure 5a depicts the film, containing CSA particles, where no reaction products were yet observed. Figure 5b presents another portion of the film, where due to film rupture and the exposure of a CSA particle to the environment, a reaction product had already formed at the edge of the flake (marked area). In this case, after water treatment of the broken composite film, the CSA particles already had access to the medium and formed ettringite. The product growth is well visible at this edge location, as well as within a crack in the film (Figure 5c), together with the “left over” from the polymer, embedded in crystallites of the hydration product (Figure 5c,d, marked locations). It is interesting to note the observation of cracks bridged by the reaction products as well (Figure 5c). The Energy dispersive X-ray (EDX) patterns for the film only (mark 1 in Figure 5c) and the hydration product (mark 2 in Figure 5d) are also given in Figure 5, bottom row, confirming the expected composition of the investigated formations, i.e., carbon only for the polymer film and calcium sulfoaluminate together with carbon content (from the surrounding film) in the case of the hydration product.

The observations discussed in this section confirm the possibility that PEO-b-PS can “interact” with cementitious hydration products in a desired direction and were for purely illustrative purposes, rather than being elaborated with tests, which is not the subject of this contribution. What was also concluded is that a PEO-b-PS composite would be sufficiently brittle to “release” the embodied load, on the one hand. On the other hand, although the PEO-b-PS composite film cannot be directly compared to nano-sized, self-assembled formations such as micelles and vesicles, the results also show that PEO-b-PS would be stable in a cement-based material and react to external and designed triggers or damage.

### 5.2. Micelles and Vesicles in (Reinforced) Cementitious Materials—Brief Review of the Main Outcomes

The concept of the approach presented in Section 4.2 was studied in sequential steps of tests in model medium, plain (not reinforced) and reinforced cement-based materials. As mentioned above, PEO-b-PS micelles and vesicles were studied. The choice of using these architectures was initially based on previous studies of nanocomposite galvanic coatings, where PEO-based nanoparticles, added to electrolytes for Zn and ZnCo electrodeposition, were found to significantly increase the corrosion resistance of the coatings [65,66]. Later on, and in view of the increasingly expressed safety and health concerns regarding the application of nanomaterials in general [67], but also in view of the concepts for built-in recyclability in the construction industry, PEO-based nanoparticles were chosen, which are otherwise considered for medical and bio-medical applications, [64,68,69,70] (i.e., no health hazards related). PEO-b-PS micelles and vesicles were used for the purpose of the above discussed concept and approach. It is well known that polymeric micelles and vesicles present numerous possibilities to alter the properties of various material classes and are used in various applications. A thorough and indeed comprehensive review on polymeric vesicles, their preparation and responsive behavior can be found in a recently reported work [71], where the application of vesicles was discussed to reach beyond the traditional field of application, biomedicine, extending to nanoreactors [70,72], perfume containers [73], catalysis [74], water remediation [75], etc. This is due to the stimuli-responsive behavior of polymer vesicles, considered as smart materials. Traditional external triggers for targeted responsive behavior are pH, temperature, light, electrical or magnetic fields, but also oxi-redox reactions (electron and ionic flows), micromechanical stress, etc. [76,77,78,79]. Hence, the applications for vesicles, together with the many possibilities to functionalize their characteristics so that the designed properties and response of the materials are achieved, are constantly increasing.

For the purpose of the concept and approach as discussed in Section 4, and in order to investigate the effects of the polymer itself on the material properties of the steel and cement-based matrix, the studies employed micelles first. Next, “loaded” nanoparticles, i.e., vesicles, were used to study degradation control with the added value of self-healing due to active substance release, which was the original aim of the study. The full scale of each experiment, together with detailed reports on the observed behavior and results are reported as separate works in specialized (corrosion or civil engineering) journals [56,57,58,80,81,82,83]. The significant effect of a very low concentration of nanoparticles (0.024 wt % per cement weight) on the global bulk matrix properties—twice lower porosity and three orders of magnitude lower permeability of the micelle-modified matrix—is also reported [80,81,84]. Compared to the (generally) employed amounts of polymers (between 0.3% and 30 wt %) and inorganic nanoparticles (e.g., Fe-, Ti-, Si-based oxides), the nanoparticles for the above discussed approach were used in extremely low concentrations (in the range of 2.4 × 10^−3^ wt % for micelles and vesicles in model aqueous medium and 6 × 10^−3^ wt % per mortar weight for micelles/vesicles in cement-based, solid systems). Therefore, a “self-repair” or “self-healing” of the product layer on steel solely due to the released Ca-component is not realistic in view of these minimal concentrations. The most plausible mechanism(s) would be linked to enhanced chloride binding effects, on the one hand. On the other hand, the nature of the incorporation of the nanoparticles in the product layer on the steel surface, adsorption on the active (anodic) areas, altered oxi/redox reactions, and/or subsequently alerted composition of the product layer on the steel surface, would be of equally large significance. The former effects on the bulk matrix properties and enhanced chloride binding are related to the increased chloride threshold, i.e., delayed corrosion initiation or unsustained corrosion propagation. These were found to be related to micro-mechanical and microstructural alterations, e.g., a more uniform distribution of low density C-S-H, hence superior global bulk matrix properties were achieved [80,81,84]. The latter effects, related to enhanced corrosion resistance, were justified by electrochemical tests in model medium [56,57,83], in cement paste and in reinforced mortar [56,58,80,84], together with results from the steel surface analysis and the steel/cement-paste interface [81,82]. The next section contains some of the latest results for the electrochemical performance of steel, together with microscopic observations. These are well in line with the above outcomes and constitute the evidence for the concept and approach presented in this work.

### 5.3. The Effect of Vesicles on the Corrosion Performance of Steel and on Bulk Matrix Properties

The utilization of nanoparticles targeted a simultaneous improvement of the electrochemical performance of the steel and the bulk matrix microstructure. Further, a possible self-healing mechanism was aimed at, specifically when Ca-based vesicles were used. The results of the application of both “empty” and Ca-bearing vesicles (PEO_113_-b-PS_760_-based) are presented here as supporting evidence for the already discussed concept and approach.

**Figure 6:**
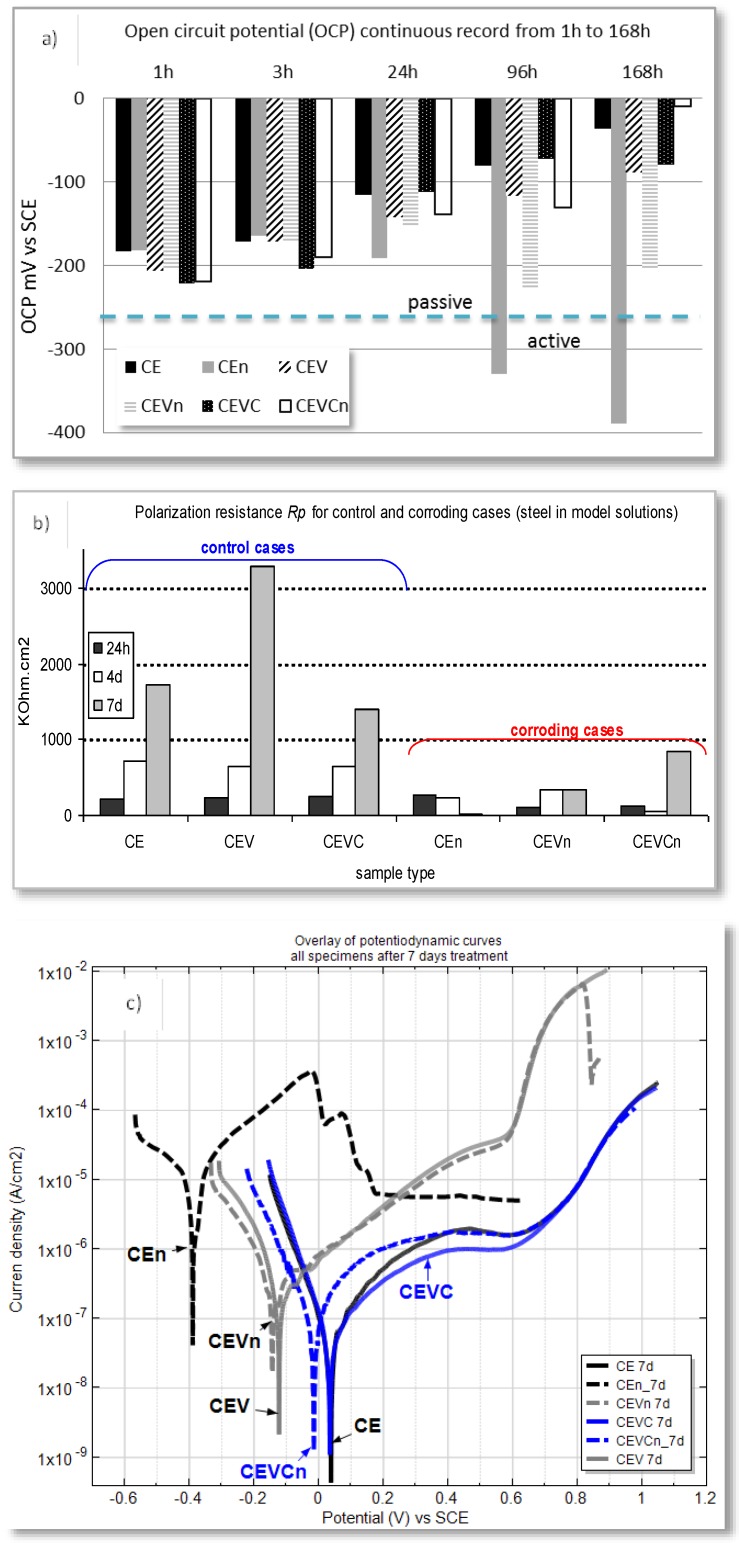
Electrochemical parameters derived for steel electrodes in model solutions: (**a**) Open circuit potential OCP records; (**b**) Polarization resistance Rp values and (**c**) Potentio-dynamic PDP response (specimens designation: CE—control, non-corroding; CEn—corroding: CEV—control, empty vesicles; CEVn—corroding, empty vesicles; CEVC—control, Ca-bearing vesicles; CEVCn—corroding, Ca-bearing vesicles).

Electrochemical measurements of steel electrodes, treated in both nanoparticle-free medium and model medium containing nanoparticles, were performed at defined time intervals after open circuit potential (OCP) stabilization. The model medium was also chloride-free (to represent control cases) and chloride-contaminated (for corroding cases). The OCP records are an indication of the active or passive state of the steel reinforcement. Figure 6a) depicts the recorded OCP values for all specimen groups in the model medium of cement extract (CE) (specimen designation and details on medium composition are as given in the supporting information). Figure 6b presents the calculated polarization resistance values (R_p_) for steel electrodes treated in all investigated solutions after 24 h, four days and seven days. As can be seen from the plots, a trend to OCP ennoblement (Figure 6a) and increasing R_p_ (increased corrosion resistance), (Figure 6b), was observed within treatment for all control cases, irrespective of the presence or absence of nanoparticles in the medium (CE, CEV and CEVC, where CE is nanoparticle-free; CEV contains empty vesicles in the medium and CEVC contains calcium-containing vesicles in the medium). This is a general outcome within the stabilization of the steel passive layer in a chloride-free, alkaline medium such as CE.

For the corroding cases (CEn, CEVn and CEVCn), the cathodic shift of the OCP values (increased corrosion activity), specifically after 24 h (Figure 6a), together with significantly lower R_p_ values (Figure 6b) were an expected outcome. An exception, however, was the CEVCn specimen, which represents steel treated in chloride-contaminated CE in the presence of Ca-bearing vesicles. Prior to 24 h, the OCP values for this specimen maintained levels similar to the active specimens CEn and CEVn (ca. −200 mV), after which an anodic shift was observed and the OCP of the CEVCn specimens ended up being even more anodic than the control case CE at the end of the seven day test (168 h). This result clearly indicates the effect of Ca-bearing vesicles towards restored steel passivity. A similar effect was not observed when “empty” vesicles were present (see OCP records for the CEVCn and CEVn specimens, Figure 6a), although the OCP for the CEVn specimens (empty vesicles) was more anodic when compared to steel treated in vesicle-free solution (CEn specimen). The above accounts for a “barrier” effect only of the polymer (vesicles themselves) for CEVn specimens, rather than altered oxidation/reduction reactions towards improved steel surface layer properties, and repair, as obviously related to the CEVCn specimens. In support of the above, the following more specific points can be noted regarding the results in Figure 6a:

(i) After 1 h and 3 h of treatment, the corroding CEn specimens (vesicle-free medium) presented OCP values in the range of those for the control CE (ca. −180 mV). For CEn specimens, corrosion initiation occurred between 3 h and 24 h and was sustained (and propagated) towards 96 h and 168 h. This is proven by the recorded cathodic OCP shift after 24 h, reaching ca. −400 mV towards the end of the test. For CEn at the initial time intervals (1 h and 3 h), corrosion initiation and propagation compete with passive layer formation in the alkaline medium, while with treatment, the reaction mechanisms as previously described in Section 2.2., were determined by the rate of chloride ion adherence, surface layer dissolution and passivity breakdown;
(ii) For the time intervals of 1 h and 3 h, the specimens in both the corroding and control conditions, where vesicles were present (i.e., CEV, CEVn and CEVC and CEVCn), initially exhibited more cathodic OCP values (between −200 and −230 mV). This is due to the competitive mechanisms of passive layer formation in the alkaline medium and the effect of vesicles and/or chloride ions. In other words, when vesicles were present, these acted as a barrier towards both passive layer formation and chloride-induced corrosion. The vesicles induced a resistance polarization for the oxidation and reduction reactions on the steel surface;
(iii) For the case of steel treated in chloride-free, empty-vesicle-containing solution (CEV specimens), surface stabilization was gradually achieved towards 168h of treatment. However, the final OCP values were not as noble as those for the control (vesicle-free) CE case (ca. −30 mV for CE and ca. −70 mV for CEV). This result is due to the abovementioned limitations, which are not relevant for CE specimens. There was only supportive evidence for barrier effects and resistance polarization for the CEV specimens; the CEV specimens also had the highest global Rp value, recorded at the end of the test (Figure 6b). The empty vesicles in the corroding CEVn specimens initially induced the same barrier effect regarding passive layer formation, but also exerted a delay in corrosion initiation. This is evident from the ennoblement of OCP from 1 h to 24 h (ca. −200 mV to ca. −180 mV). After 24 h, however, the OCP for CEVn specimens shifted in the cathodic direction, reaching approx. −240 mV after 96 h;
(iv) In contrast to all above cases, the steel electrodes in the medium with Ca-bearing vesicles (CEVC and CEVCn specimens) present similar values at 1 h and 3 h, irrespective of the presence of chloride ions in the medium. These values are more cathodic, accounting for limitations regarding passive layer formation. For the control case (CEVC) an anodic shift was observed around 168 days, similar to the CE and CEV groups. Contrary to the corroding cases (CEn and CEVn) discussed above, the corroding specimens (CEVCn) show ennoblement only at all time intervals, with the most noble OCP at the end of the test. This accounts for a restructuring of the passive film on the steel surface for CEVCn, most likely the formation of a Ca-substituted product layer with higher corrosion resistance (phenomena previously discussed in Section 2). The superior corrosion resistance for CEVCn specimens was obviously triggered by the Ca-bearing vesicles in this case.

The OCP evolution discussed above for all specimens is well in line with the derived Rp values (Figure 6b). For instance, the Rp values for the CEVCn specimens clearly show an increasing trend, i.e., improved corrosion resistance towards the end of the test (Figure 6b), which was not observed for CEVn specimens, where “empty” vesicles were employed. Here again the difference between CEVn and CEVCn specimens is to be attributed to the vesicle type in the medium—“empty” for the former and Ca-bearing vesicles for the latter case—where the Ca-bearing vesicles introduce superior product layer properties to the steel surface.

The higher corrosion resistance and improved properties of the product layer in CEVCn specimens, potentially due to the “repair” of the initial corrosion damage, is evident from the potentio-dynamic PDP response for all cases at the end of the test, Figure 6c.

After seven days of treatment, the corrosion and anodic currents for the corroding CEVCn specimens were comparable to those of the control (non-corroding) CE and CEVC cases, where the corrosion current for CEVC was the lowest recorded. In contrast to the altered electrochemical state of the steel surface in the presence of Ca-bearing vesicles, barrier effects alone are the most plausible for the steel specimens treated in “empty” vesicle-containing medium. This is evident from the similarly higher corrosion and anodic currents for both control (CEV) and corroding (CEVn) specimens (Figure 6c, CEV and CEVn).

In other words, corrosion propagation for the case of CEVn was impeded, while the establishment of a corrosion-resistant passive film for CEV specimens was also inhibited. The resulting currents were approximately one order of magnitude higher for these cases, compared to the control CE and the corroding CEVCn specimens. The highest corrosion and anodic currents were recorded for the corroding CEn specimens (Figure 6c), which was as expected for steel treated in nanoparticle-free, chloride-containing medium.

In view of the corrosion resistance and electrochemical-method-derived performance for steel in a cementitious matrix, it would be expected that the steel/cement-paste interface would have different properties in the presence of admixed nanoparticles. This was as previously discussed in relation to the concept and approach of nanoparticle application and with regard to Figure 1, where the relevant interfaces were schematically presented. Figure 3, as previously discussed, depicts micrographs of the steel/cement-paste interface in control and corrosion-reinforced mortar specimens, where the matrix was not modified with nanoparticles. As can be observed, an intact interface is relevant for the control case (Figure 3a), while corrosion products are clearly visible for the corroding case (Figure 3c), already penetrating the bulk matrix at a distance of more than 200 micrometers away from the steel surface. Figure 3 is an illustration of the reason for the steel/cement-paste interface degradation, and ultimately the reinforced concrete degradation, when chloride-induced corrosion takes place. For the corroding specimen (Figure 3c), corrosion products such as Cl-containing iron oxi/hydroxides formed on the steel surface (e.g., akaganeite, Figure 3d). These were volume-expanding and caused continuous micro-cracking in the restricted bulk volume of the cement paste material. In contrast, for the control and non-corroding conditions, the steel surface was generally covered by CaO/Ca(OH)_2_-substituted product layers, see Figure 3b, which except for maintaining steel passivity, did not cause microstructural alterations of the bulk matrix.

**Figure 7:**
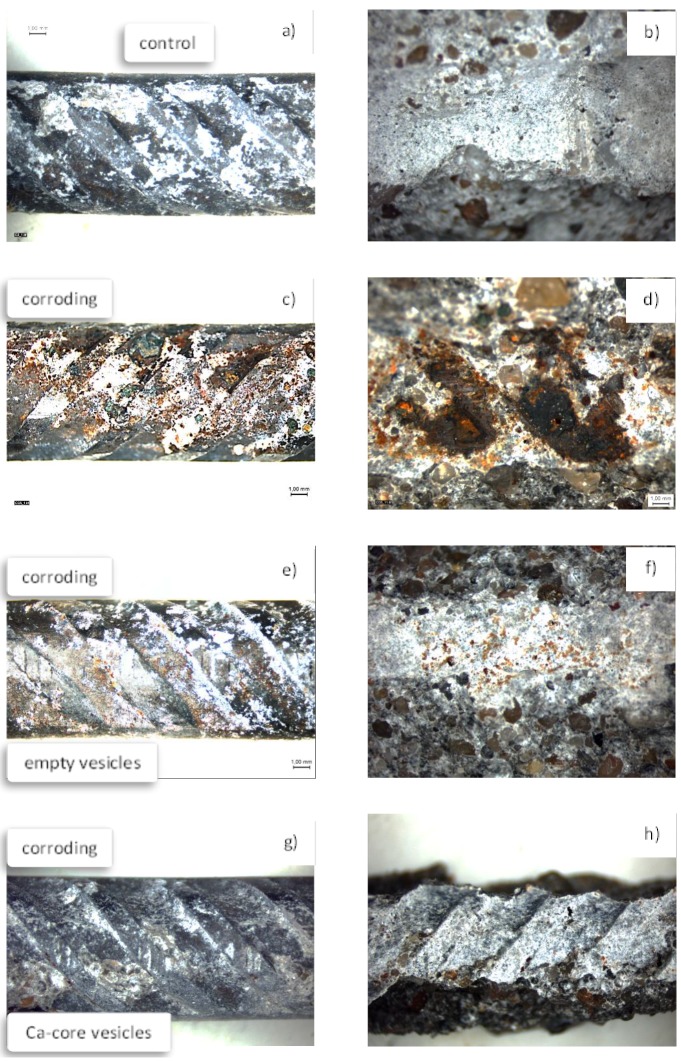
Light microscopy of the steel reinforcement (left column) and the corresponding “print” in the cement paste (i.e., adjacent bulk matrix) for control and corroding, vesicle-free specimens (**a**–**d**) and corroding, vesicle-containing specimens (**e**–**h**). The observations were recorded after the specimens were broken open after 300 days of conditioning.

In the presence of vesicles and upon corrosion initiation, a re-structuring of the product layer on the steel surface was expected, following the mechanisms described in Figure 1. A product layer rich in Ca-substituted iron oxi/hydroxides is relevant for a matrix modified with Ca-bearing vesicles (as indirectly determined by electrochemical tests). In contrast, at minimum, barrier effects and reduced corrosion product formation should be observable for the matrix modified with “empty” vesicles. These outcomes were in fact recorded for the steel/cement-paste interface in the presence of both vesicle types and this was supported by X-ray analysis on the steel surface. A detailed analysis of all conditions and the complete investigation of the reinforced mortar is reported in detail in [82]. In this contribution, the following supportive evidence (Figure 7) is briefly discussed based on light microscopy on the steel surface and the adjacent cement paste, illustrating the main objective of this work, i.e., presenting the concept of the approach utilizing nanoparticles for reinforced-cement-based materials. 

As can be observed in Figure 7a,b for the control condition, there were no corrosion products on the steel surface and no corrosion products deposited in adjacent to the steel cement paste. Similarly, no corrosion products were observed for the specimens where the mortar contained Ca-bearing vesicles (Figure 7g,h).

In contrast, corrosion product accumulation on the steel surface and penetration into the bulk matrix were observed for the corroding, vesicle-free specimen (Figure 7c,d). When “empty” vesicles were admixed in the mortar matrix, the previously discussed limitations towards both passive layer formation, but also impeded corrosion propagation, resulted in reduced corrosion product formation, however, with evidence of corrosion product penetration into the bulk matrix (Figure 7e,f). Hence, the previously discussed corrosion resistance of the steel electrodes in simulated medium, recorded via electrochemical measurements (Figure 6), and the hypothesized performance in view of the steel/cement-paste interface, are hereby visualized (Figure 7) and evidence is provided of the “self-healing” or “self-repair” of the product layer on the steel surface in the presence of Ca-bearing vesicles.

The concept of nanoparticle application for reinforced concrete, as discussed in Section 4, clearly emphasized the importance of the approach in view of simultaneously affecting the steel reinforcement and the bulk matrix in a reinforced concrete system. The effect of nanoparticles on the bulk matrix properties was thoroughly investigated, starting from less heterogeneous systems (cement paste, plain mortar) and later studying reinforced-cement-based materials. Microstructure (e.g., porosity, pore size), micromechanics (e.g., elastic modulus of low and high density CSH), compressive strength, permeability, etc., were recorded for micelle- and vesicle-free mixtures versus modified mixtures. Some of the these results were briefly mentioned in Section 5 and reported in detail in specialized journals [80,81,82,83,84].

**Figure 8:**
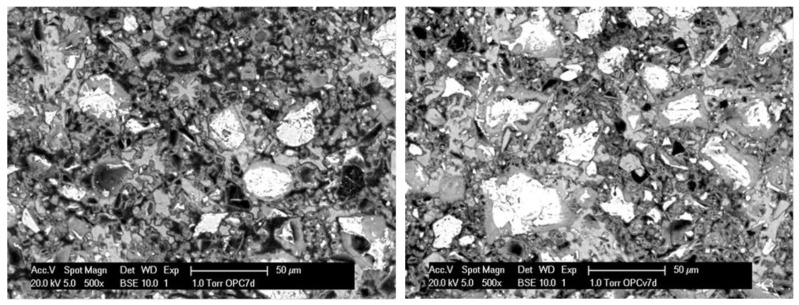
Cement-based bulk matrix (impregnated and polished cross sections of mortar specimens, as usually used for ESEM image analysis) without (**left**) and with (**right**) admixed nanoparticles (vesicles) at seven days of hydration age (black color represents pores and voids in the matrix, white color represents un-hydrated phases (cement grains), the grey colored features represent hydration products (e.g., C-S-H, Ca(OH)_2_; for image analysis of the bulk matrix, aggregates (as sand, gravel and relevant interfacial transition zones ITZs) are to be excluded, therefore the images do not present aggregates or interfaces).

As supporting evidence of the concept and approach of nanoparticle application, the subject of this work (Figure 1), Figure 8 depicts the bulk matrix of mortar specimens at the hydration age of seven days as a comparison of a vesicle-free matrix (Figure 8 left) and Ca-based, vesicle-containing matrix (Figure 8 right). Of course, without quantification and detailed analysis of images such as those in Figure 8, no claims or scientific judgment can be made. Moreover, microstructural analysis is generally relevant to at least 35 locations (images of magnification 500×, such as the ones in Figure 8). This is performed for statistical accuracy and following a known methodology that has been reported in detail [18,63]. Therefore, no further discussion will be included here, but rather pointing out only the clearly observable difference in the images in Figure 8.

As can be seen, the vesicle-free specimen (Figure 8, left) presents a relatively higher level of pore and void distribution, while the matrix in the vesicle-modified specimen (Figure 8, right) depicts well-visible high and low density CSHs (and less pores and voids). This illustrates the positive effect of admixed vesicles, as targeted and previously discussed within the concept of the approach in Section 4. These outcomes are in line with what was reported for the effect of PEO-containing polymers on the properties of fresh cement paste, where reduced coagulation and sedimentation of the cement particles was discussed as being the result of a polymer-induced increase in electrostatic or repulsive forces during cement hydration [85,86]. The result is an altered distribution of the hydration products in the matrix, as also seen in Figure 8 and previously reported for both micelles and vesicles [81,84], where in addition, the aforementioned “nucleation site effect” [24] contributes further to the reduced porosity and permeability of the bulk cement matrix.

## 6. Conclusions and Outlook

The purpose of this contribution was to present the concept of an approach to employ polymeric nanoparticles to control reinforced concrete degradation. 

The originality of the approach to control reinforced concrete degradation via specific nanoparticles is in the targeted simultaneous improvement of both steel and concrete properties, prior to any degradation, and to repair damage later on, in the event of impaired properties. The advantage of the utilization of nanoparticles in the discussed manner, is that these particles, initially introduced in the system to improve the mechanical and microstructural performance of the bulk cementitious material and the steel/cement-paste interface, will further participate in a self-healing process in terms of corrosion protection or product layer repair on the steel reinforcement.

Conclusive statements can be made on two main aspects: the implementation of tailor-made nanoparticles and autonomous self-healing. The former (tailored particles) have so far proven to be a feasible approach for corrosion control, evidenced by the significant influence of the minimal concentration of these on the material properties, namely achieved corrosion delay, superior steel product layer characteristics and contribution to increased passivity, rather than only enhanced barrier effects. The latter (self-healing aspects) are justified by (at the very least) the corrosion performance of steel when in contact with “empty” or Ca-bearing nanoparticles. For the former case, corrosion delay is only relevant, but no evidence of an improved electrochemical response was observed. For the latter case, corrosion propagation was not observed, which together with the improved stability of the product layer after an initially more active state, shows the possibility for self-healing or self-repair on the steel surface.

The next step in view of the above approach is to study the local electrochemical response on the steel surface (at the nano- to micro-levels), together with (changes in) the micromechanical properties of the product layer versus an altered chemical composition and morphology of the nanoparticles themselves and upon designed triggers. These results, when linked with the already available outcomes on global corrosion performance and the microstructural characteristics of the cement-based material and interfaces, are expected to substantiate the approach even further. For instance, the results will allow a simulation approach to provide evidence for this concept of corrosion control in view of the predictions regarding material performance versus optimum design by, e.g., varying the chemistry and concentration of the nanoparticles. Ultimately, this will support the feasibility of practical applications in reinforced concrete structures, with relevance to cost justifications and service-life predictions, compared to existing and more conventional methods and practices.

## Supporting Information: Materials and Methods

### Model Medium and (Reinforced) Cement-Based Systems

Model aqueous medium (cement extract) was used as a simulated pore solution for preliminary tests on the corrosion performance of steel in the presence of nanomaterials. Cement paste, mortar and reinforced mortar were studied as the solid, cement-based materials. The CE was prepared by mixing Ordinary Portland cement (OPC) CEM I 42.5N and water in a ratio of 1:1, followed by stirring the suspension for 24 h and subsequent filtration. The pH of the filtrate (i.e., the CE) was 12.7, and the chemical composition (derived by inductive coupled plasma spectrometry, ICP) was: Ca—201 mg/L; K—3.85 mg/L; Na—1.33 mg/L; Al—4 mg/L and Fe—<1 mg/L. Both chloride-free and chloride-containing CE were used, and NaCl adjusted to 1% NaCl in the CE was used as a corrosion-accelerating medium. These solutions were also additive-free or contained additives (i.e., polymeric nanomaterials and/or NaCl were added to the solution according the test requirements). More details on this generally employed methodology to test the corrosion performance of steel in CE, as a simulated environment, can be found in [51,52,56,83].

Plain (non-reinforced) mortar cubes were cast to evaluate the properties of the cementitious bulk matrix, while reinforced mortar cylinders were used for tests of the steel corrosion state, cement-based matrix properties and steel/cementitious materials interface. Identical cement type (OPC), water-to-cement ratios (w/c 0.5) and cement-to-sand ratios (c/s 1:3) were used for all cement-based specimens. The non-reinforced-cement-based specimens were studied in sequential steps, e.g., after three, seven, 14 and 28 days of cement hydration for deriving microstructural and mechanical properties. This is a general approach in concrete material science to evaluate properties and performance with time of cement hydration. Details on all these types of experimental series are reported in [53,54,55,56,57,58,80,81,82,84].

After casting, the reinforced mortar cylinders with centrally-embedded reinforcement were cured in a fog room (20 °C, 98% relative humidity) for 28 days. The specimens were lab conditioned as 1/3 immersed in water or 5% NaCl solution as external medium for the full test duration (>250 days). This cylindrical geometry and specimen handling and conditioning is also as usually employed in corrosion studies on reinforced mortar and concrete, as reported in detail in the references cited above.

### Steel Electrodes and Steel Reinforcement

For the tests in CE, steel electrodes (St37) with a surface area of 4 cm^2^ were used, while construction steel FeB500 HKN (with a surface area of 16 cm^2^), centrally embedded in mortar specimens, were evaluated in reinforced mortar. All steel bars and St37 electrodes were equally treated prior to conditioning in the relevant solutions and/or casting in the mortar specimens (grinding, polishing, acetone cleaning and water rinsing for St37, and acetone cleaning and water rinsing for FeB500 HKN prior to casting). Three replicates per environment and condition were tested. The sample designation, relevant to the results discussed in this work, was as follows: group CE—control cases (chloride-free and nanoparticle-free); CEn—corroding cases (NaCl-containing medium); CEV and CEVC stand for the non-corroding cases of steel in CE and mortar, where “empty” or Ca-loaded nanoparticles were present (either in the external CE medium or admixed in the mortar mixture), whereas CEVn and CEVCn stand for the corroding, nanoparticle-containing cases, respectively.

### Nanoparticles (Micelles and Vesicles)

The nanoparticles in this work—micelles and vesicles—were polyethylene oxide polystyrene (PEO-b-PS)-based, where the copolymer was synthesized by atom transfer radical polymerization (ATRP) employing the macroinitiator technique [87]. PEO_113_-b-PS_70_ di-block copolymer was used for the micelles, while PEO_113_-b-PS_760_ was used for vesicle preparation. Both the micelles and vesicles were obtained by the dialysis method. The aqueous solution of micelles (or vesicles) in a concentration of 0.5 g/L, was added to the aqueous model medium (CE) or used directly as mixing water for the cement-based systems, resulting in 0.0024 wt % of the nanoparticles in aqueous medium or 0.024 wt % per cement weight in the solid (cement-based) systems. Micelles and empty vesicles were employed first, in order to study material performance when no self-healing-related mechanisms were involved. In order to distinguish between “barrier” effects and “self-healing” due to Ca-release, both “empty” and Ca-containing vesicles were further used. Dynamic light scattering (DLS) (Malvern zeta-sizer, Nano ZS90, NL) and transmission electron microscopy (TEM) (Jeol JEM 1400 TEM) were performed, confirming a hydrodynamic radius of 50 nm for the micelles and 220 to 250 nm for the vesicles. Full details on the micelle and vesicle preparation and characterization are as previously reported in [80,81,82,83].

### Electrochemical Methods and Microscopy

A Potentiostat PGSTAT 302N (Metrohm, NL) was used for all electrochemical tests for both steel in CE and for reinforced mortar. A common three-electrode cell arrangement was used for CE, where a saturated calomel electrode (SCE) was used as the reference electrode, Pt was used as the counter electrode, and a steel electrode was used as the working electrode. The geometry of the reinforced mortar cylinders was also designed to resemble a three-electrode cell, where external Ti mesh served as the counter electrode, the centrally embedded reinforcement was the working electrode and an external SCE was the reference electrode (full details on this generally employed cell arrangement can be found in [53,54,82]). Linear polarization resistance (LPR) was performed in the range of ±20 mV vs OCP to derive polarization resistance (R_p_) values, using linear regression and well-known considerations [88,89,90]. Potentio-dynamic polarization (PDP) was employed in the range of −0.2 V to +1.0 V vs open circuit potential (OCP) at a scan rate of 0.5 mV/s. The PDP curves allowed comparison of the electrochemical response with external and prolonged polarisation in view of the resistance to the anodic polarisation of the product layer on the steel surface (all electrochemical measurements were as generally employed for systems such as those in this study and as reported in the works referenced above).

Scanning electron microscopy (SEM) was used for morphological and microstructural studies, using an environmental ESEM Philips XL30, equipped with an energy dispersive X-ray (EDX) detector. SEM studies were relevant to the electrodes treated in CE steel, the steel reinforcement, the cementitious bulk matrix and the steel/mortar interface (experimental details and results for the complete test series can be found in the previous reports cited above). Light microscopy was performed as well and included in this work for visualization purposes, supporting the concept and feasibility of the discussed approach.
